# The Effect of Digital Platform Strategies on Firm Value in the Banking Industry

**DOI:** 10.1080/07421222.2024.2340825

**Published:** 2024-06-24

**Authors:** Maximilian Schreieck, Yongli Huang, Alexander Kupfer, Helmut Krcmar

**Affiliations:** a University of Innsbruck, Innsbruck, Austria; b Technical University of Munich, Munich, Germany

**Keywords:** Digital platform strategies, digital platform ecosystems, open banking, banks, emerging markets, AI orientation, APIs, event study

## Abstract

After digital platforms have become successful in the information technology (IT) industry, incumbents from traditional industries increasingly implement digital platform strategies. However, there is mixed evidence on whether these incumbents benefit from digital platform strategies. To provide systematic insights, we focus on the banking industry. With the emergence of open banking, banks have begun implementing digital platforms to unlock the innovative power of third-party developers. We conducted an event study based on the announcement of digital platform strategies in a global sample of 165 banks. We show that, on average, investors react positively to the announcements. Contrary to our expectations, this effect is more substantial for banks from emerging markets than those from developed markets. Prior artificial intelligence (AI) orientation only partly contributes to investors’ favorable perception of a digital platform strategy. These results point to the complex interplay of AI orientation and digital platform strategies, yielding questions for future research.

## Introduction

Digital platform strategies are on the rise—not only in information technology (IT) companies but also in companies from traditional industries such as banking, insurance, and manufacturing [80, 82, 93]. Digital platforms form the core of digital platform ecosystems in which third-party developers generate apps and services for the platform owner’s customers [74, 96, 97]. Digital platforms foster generativity by providing application programming interfaces (APIs) and other resources that third-party developers can use to develop apps and services [18, 89, 101, 105]. Network effects further fuel the growth of digital platforms as third-party developers’ apps and services attract customers. These customers, in turn, make the platform more attractive for further third-party developers [18, 105].

While an increasing number of incumbent companies from traditional industries are implementing digital strategies, there is mixed evidence of whether these strategies lead to increased performance [2, 30]. Although several case studies indicate that digital platform strategies create opportunities for incumbents [82, 84], these and further studies highlight the challenges such a strategy creates [84, 93].

To provide more systematic results on the potential of digital platform strategies for incumbents in traditional industries, we conducted an event study in the banking industry, analyzing the investors’ reaction to incumbent banks’ announcement of a digital platform strategy. We chose the banking industry as the context for this study because compared to other traditional industries such as manufacturing, health care, or agriculture, the share of companies that have implemented a digital platform strategy is higher [23]. Furthermore, banks have a more homogenous business model across companies and regions, and we consequently expect to observe a more homogenous effect of announcing a digital platform strategy. In the banking industry, a digital platform strategy refers to providing open APIs that allow third-party developers, such as fintech companies, to access the data of the banks’ customers to create additional apps and services for them. This approach is also referred to as open banking [9, 13].

We focus on the investors’ reaction to the announcement of digital platform strategies because this immediate reaction is better suited to capture the effect of the strategy than performance measures available annually. Performance measures that directly capture the generativity of the digital platform, such as the number of apps, the number of app updates, and API usage, are not available publicly for most banks. Furthermore, the performance impact of a digital platform strategy might take effect with a time lag of unclear length, making the investors’ immediate reaction a measure that can be captured more consistently across banks.

Despite this rise in digital platform strategies in the banking industry, there are only anecdotal insights into whether these strategies contribute to the banks’ performance. The aim of this study is thus to extend this limited knowledge by examining investors’ evaluations regarding digital platform strategies. Hence, we pose the following research question:

Research Question: How do investors react to the announcement of a bank’s digital platform strategy?

To provide a nuanced answer to this question, we are further interested in two moderating factors on the effect of digital platform strategies. First, we analyze whether banks from developed markets see a more substantial positive impact of a digital platform strategy than banks from emerging markets because they have access to more third-party developers in their established domestic market, creating stronger network effects than their counterparts in emerging markets.

Second, we study whether banks with a higher orientation toward artificial intelligence (AI) can leverage a digital platform strategy better than banks without such an AI orientation. We hypothesize that AI orientation helps banks foster generativity on their digital platform because third-party developers can leverage AI technologies in innovative apps and services.

For the event study, we constructed a global sample of 165 banks. Assuming that financial markets are efficient [65], we examine investors’ responses to the announcement of a digital platform strategy by capturing abnormal stock market returns. First, we find that the announcement of a digital platform strategy is associated with significant positive abnormal stock returns. Second, we examined the moderating effects by subsampling our sample in banks from developed vs. emerging markets and banks that have displayed AI orientation vs. banks that have not.

The respective subsample analyses provided evidence that contrary to our expectations, banks from emerging markets benefit more from a digital platform strategy. The subsample analysis on AI orientation only partially confirmed our assumption that AI orientation contributes to investors’ positive reactions to a digital platform strategy. However, a post hoc analysis that zooms in on the sub-subsamples suggests that AI orientation benefits banks from emerging markets. In contrast, its effect on banks from developed markets is inconclusive.

With these findings, we add to the literature on digital platforms by observing that investors generally react positively to the announcement of a digital platform strategy in more traditional industries such as banking. Given that previous literature has highlighted both the opportunities and challenges of digital platform strategies in traditional industries, our findings indicate that the potential outweighs the risks from the investors’ perspective.

By considering a global sample of banks, we address current concerns that emerging markets are understudied in information systems [11, 54]. Analyzing the impact of digital platform strategies only in developed markets would neglect the opportunity to understand better if banks from emerging markets can also benefit from digital platforms. Indeed, we found that investors react more positively to banks from emerging markets announcing digital platform strategies, even more so if they display AI orientation. These findings suggest that investors value the growth potential of these banks more than the easier access to network effects in developed markets.

## Background

As background for the hypotheses development, we lay out generativity and network effect as crucial mechanisms in digital platform ecosystems. We then review information systems literature on digital platforms in traditional industries, and explain why we chose the banking industry as the context and the investors’ view as the dependent variable in our study.

### Generativity and Network Effects in Digital Platform Ecosystems

**Figure 1.**
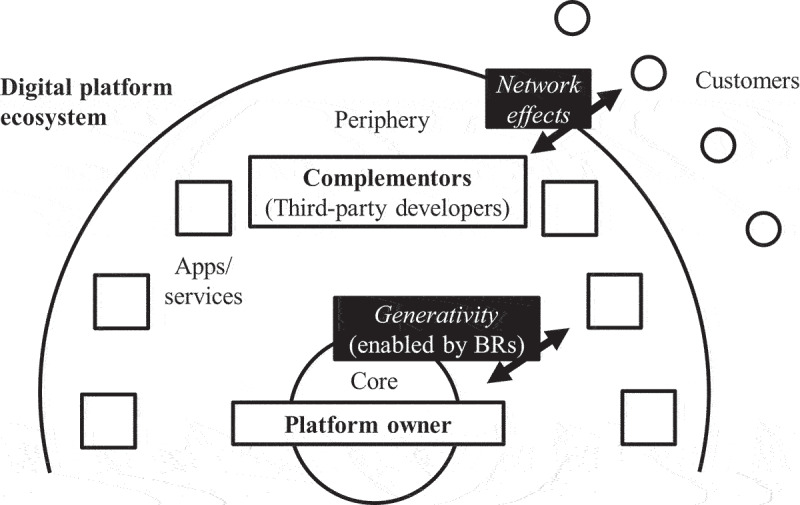
Digital Platform Ecosystems; adapted from Tiwana [96]; BRs: boundary resources.

Digital platforms—defined as the “extensible codebase of a software-based system that provides core functionality shared by the modules that interoperate with it” [96, p. 676]—form the core of digital platform ecosystems (Figure 1). In these digital platform ecosystems, complementors (i.e., third-party developers) implement apps and services drawing on the platform’s boundary resources such as APIs, software development kits (SDKs), documentation, blueprint implementations, and developer communities [74, 89, 97].

Generativity and network effects fuel the growth of digital platform ecosystems (Figure 1). First, the digital platform enables generativity by third-party developers [18, 105]. By providing boundary resources the digital platform supports third-party developers in creating high-quality apps and services [89]. As a result, potentially innovative apps and services emerge in the periphery of the digital platform ecosystem, shifting the locus of innovation from the inside to the outside of a company. This change is also referred to as “inverting the firm” [74, p. 255]. Digital platforms that draw on third-party developers’ apps and services to generate innovation are also referred to as innovation platforms, as opposed to transaction platforms that focus purely on matching and connecting transaction partners (e.g., Airbnb, Uber) [25].

Second, once apps and services are available on the digital platform, network effects contribute to the further growth of the ecosystem [12, 55, 73]. The apps and services available on the digital platform attract customers, and the more customers use the platform, the more attractive it becomes for third-party developers to offer new apps and services. This type of network effect is also referred to as a positive cross-side or indirect network effect [90].

A digital platform strategy refers to implementing a digital platform to create a flourishing ecosystem by leveraging generativity and network effects as growth mechanisms. The success of digital platform strategies is most visible in the IT industry. For example, Apple and Google have established digital platforms for mobile apps with their smartphone operating systems [25]. These big tech companies have fostered generativity in their ecosystems by providing powerful development tools, including developer portals, software development kits, and strong communities. As of 2022, 1,768 million apps were available for Apple’s iOS platform and 2,673 million for Google’s Android. Network effects were crucial for the growth of both ecosystems as increasing numbers of apps attracted customers and vice versa.

### Digital Platform Strategies in Traditional Industries

Digital platform strategies have spread from the IT industry to more established, traditional industries because they promise an ecosystem of innovative solutions that enhance the incumbents’ core business. Incumbents from various traditional industries have implemented digital platforms, including automotive manufacturing [85, 93], process automation [82], insurance [80], banking [103], agriculture [57], and health care [17] industries.

However, whether digital platform strategies benefit incumbents from traditional industries is unclear [cf. 2,30]. Findings from the information systems literature point to both opportunities and challenges of digital platform strategies for incumbents (Table 1).

**Table 1.** Opportunities and challenges of a digital platform strategy for incumbents.

Opportunities for Incumbents	Description	References
Value creation through new interactions	A digital platform strategy enables the generation of new knowledge and learning through new interactions and information exchanges.	Sandberg, Holmström, and Lyytinen [82]
Digital innovation	With a digital platform strategy, companies develop the capability to empower third-party developers.	Svahn, Mathiassen, and Lindgren [93]
Defense against new entrants	A digital platform strategy helps incumbents to “respond to the threat of innovative digital natives in their industry” (p. 1355).	Schreieck, Wiesche, and Krcmar [84]
Competition with dominant digital platforms	A digital platform strategy helps incumbents emancipate themselves from a complementor role on a competing dominant digital platform.	Khanagha et al. [58]
Challenges for Incumbents	Description	References
Change of organizing logic	A digital platform strategy drives complexity and leads to transitions in the company’s organizing logic.	Sandberg, Holmström, and Lyytinen [82]
Identity tensions	A digital platform strategy requires a new organizational identity, causing tensions with the inherited identity.	Lindgren, Eriksson, and Lyytinen [64]
Tensions between internal and external actors	A digital platform strategy fosters external collaboration, which challenges carefully balanced internal cooperation.	Svahn, Mathiassen and Lindgren [93]; Schreieck, Wiesche and Krcmar [85]
Ecosystem transformation	A digital paltform strategy requires moving existing partners and customers to the platform ecosystem.	Schreieck, Wiesche and Krcmar [84]

On the one hand, generativity and network effects create opportunities. As companies establish a digital platform and open interfaces to external parties, they engage in new interactions and information exchanges [82] and enable new forms of value creation that span their organizational boundaries. By doing so, incumbents develop the capability to empower third-party developers to create “digital” innovation, as opposed to traditional “physical” innovation [93]. As the digital platform becomes established, network effects kick in and strengthen the position of the incumbents in their market. This position helps incumbents to defend against new entrants—often digital native startups that aim to establish a digital platform in traditional industries [84]—or to fight back against an already dominant digital platform established, for example, by big tech companies [58].

On the other hand, implementing a digital platform strategy creates challenges for incumbents. “Inverting the firm” to unlock the generativity of third-party developers requires that incumbents change their organizing logic [82] and their organizational identity [64]. For example, the Swiss process automation company ABB had to digitize its physical product platform and establish new organizational practices and routines to enable value creation with external actors [82]. Once generativity by third-party developers occurs on the platform, tensions between external and internal collaboration can emerge [93]. For example, when BMW planned to open its digital platform for onboard apps, it first had to define the role of internal business units on the new platform to address their concerns about becoming obsolete [85]. Furthermore, incumbent companies typically have an established ecosystem of partners and customers. For a digital platform strategy to be successful, these partners and customers need to transition to the digital platform ecosystem as well—a constant challenge that requires the platform owner to demonstrate the benefits of the new digital platform ecosystem [84].

While the opportunities and challenges identified in previous qualitative studies can help incumbents avoid pitfalls, quantitative analyses on the impact of digital platforms are missing. Thus, it is unclear whether digital platform strategies present an opportunity for incumbents and under which conditions.

### Digital Platform Strategies in the Banking Industry

To further investigate the impact of digital platform strategies, we focus on the banking industry as a representative traditional industry. The banking industry is a suitable context for two reasons. First, in the banking industry, the share of companies that have announced digital platform strategies—often referred to as open banking strategies [9, 13]—is relatively high [23] because of the increasing competition by fintech startups [49, 53], new digital technologies such as AI that can enhance digital platforms with customer-facing AI solutions, and because regulation such as the European Union (EU)’s Payment Services Directive regulation (PSD2) and the United Kingdom (UK)’s Open Banking Standard [72] pushed banks to open up their core systems with digital platforms. Thus, results from analyzing digital platform strategies in the banking industry can help companies from other traditional industries, such as health care and manufacturing, where the adoption of digital platforms is lower.

Second, the traditional business model of banks is homogenous across companies and geographic regions. Fundamentally, banks address retail and corporate customers who deposit money in various deposit products and borrow money through different loan products. In addition, many banks engage in the insurance business and the trading of various financial instruments. Differences exist between banks concerning their target audience (community vs. private vs. commercial banks) or focus (savings banks vs. land development banks); however, they still share a core set of activities. This homogeneity of the business model helps isolate the effect of implementing a digital platform strategy. In industries such as health care, manufacturing, and agriculture, companies can have heterogeneous business models, focusing on specific parts of the value chain. For example, in the manufacturing industry, digital platforms might be implemented by original equipment manufacturers as part of the product they market or by suppliers as digital platforms for manufacturing in the industrial Internet of Things. In addition, the focus of companies might vary with the geographical region. In some countries, suppliers predominate, while original equipment manufacturers are central to the economy in others. In such heterogeneous settings, it is challenging to design a quantitative study that analyzes the impact of digital platform strategies.

In sum, the banking industry is a suitable context for our study. The foundations of digital platforms in banking are open APIs through which banking data are shared between two or more unaffiliated parties [42]. Typically, a digital platform strategy entails a developer portal on which third-party developers find documentation of APIs, blueprints for their implementation, answers to frequently asked questions, and opportunities to engage with other third-party developers. Table 2 illustrates a typical digital platform strategy implemented by Deutsche Bank, one of Germany’s largest financial institutions.

**Table 2.** Illustration of a digital platform strategy using Deutsche Bank’s Open API Program.

Term	Illustration
Platform owner	Deutsche Bank
Digital platform	Deutsche Bank Open API, including developer resources: 41 API and SDK products (e.g., “Age Check,” “Decoupled Wallet SDK,” and “IBAN Check”) Documentation of API and SDK products A community for developers: Deutsche Bank API Partner Network, hackathons
Complementors (Third-party developers)	Firms participating in Deutsche Bank’s Open API Program: Buhl: tool for tax declarations in Germany, automatically retrieving relevant data from the customers’ Deutsche Bank accounts Finanzguru: app for individuals to track expenses and manage accounts at different banks Madco: plugin for age verification in webshops using the customers’ Deutsche Bank information

Notes: API: application programming interfaces; SDK: software development kits; IBAN: international bank account number.

**Figure 2.**
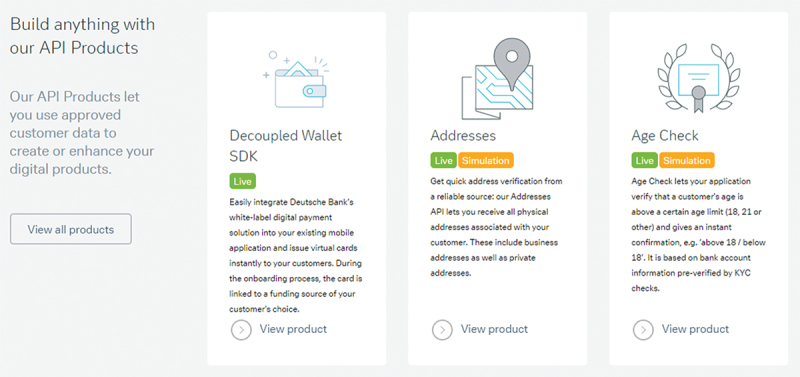
Screenshot from the Developer Portal of Deutsche Bank’s Open API Program [27]. API: application programming interfaces.

In 2017, Deutsche Bank launched its Open API Program to attract third-party developers [26]. The Open API Program grants developers access to various APIs, SDKs, and packages that help them create apps for Deutsche Bank customers. APIs, for example, provide customer data that third-party developers can use in their apps. Packages include functionality such as an age check or an international bank account number (IBAN) check that developers can easily integrate into their app (Figure 2).

Third-party developers include companies such as Buhl, a market leader in Germany for tax declaration software, who integrated the Deutsche Bank API into their product to automatically retrieve data from the customers’ bank accounts for the tax declaration. Another example is Finanzguru, a German startup founded in 2015 that helps customers track expenses and manage accounts from several banks. The Deutsche Bank API allows Finanzguru to automatically pull information on their customers’ payments. Deutsche Bank established a developer community through its Deutsche Bank API Partner Network and organized hackathons to spark generativity in the ecosystem further. The digital platform strategy was generally covered positively in investor news outlets [66].

### Investors’ View on Digital Platform Strategies

To analyze whether digital platform strategies such as the one implemented by Deutsche Bank have a positive impact, we investigate the investors’ short-term reaction to the announcement of a digital platform strategy using the event study methodology. While we are ultimately interested in the impact of digital platform strategies on the performance of banks, the investors’ short-term reaction can be seen as a proxy because they will react positively to an announcement of a digital platform strategy if they believe that it will contribute to better performance [65].

We opted to analyze the investors’ reactions for three reasons. First, the investors’ immediate reactions allow us to isolate the impact of a digital platform strategy better than financial performance measures such as return on assets (ROA) or Tobin’s Q. These measures are typically used in studies with annual observations. However, over the course of a year, different factors might have affected these measures, thus diluting the possible impact of the digital platform strategy. Similarly, measurements of the investors’ long-term reaction, for example, by analyzing the performance of stock prices over time, make it more challenging to single out the impact of announcing a digital platform strategy.

Second, between the announcement of a digital platform strategy and a potentially positive effect on performance measures is a time lag of unclear length. It has been shown for other investments in IT that the time lag of an effect on performance can range between three to seven years [14, 83]. This time lag might differ for digital platforms and vary from company to company. For example, Deutsche Bank first ran a one-year test phase of the platform after the initial announcements [26]. By analyzing investors’ immediate reactions, we avoid measuring issues caused by time lag.

Third, measures to evaluate the innovation output of digital platforms are rarely available. For digital platforms such as Google Android or Apple iOS, data on the number of apps and app updates can be crawled and used as measures for innovative performance [40]. For most digital platforms implemented by banks, these data and similar proxies, such as API usage, are not publicly available. In sum, the investors’ reaction is the best available measure to approximate the impact of announcing a digital platform strategy.

We argue that previous studies of investors’ reactions to IT-related announcements do not cover digital platform strategy.^1^ As shown in the overview in the Online Supplemental Appendix 1, Table 1.1, previous studies have analyzed investors’ responses to announcements on IT investments [28], e-commerce initiatives [91], ERP investments [79], information security investments [19], and identity theft countermeasures [11]. The announcement of a digital platform strategy differs from the above announcements because it focuses on the potential of generativity by external actors and network effects between these actors and customers rather than on the use of IT internally.

## Hypotheses Development

Building on the theory of generativity and network effects in digital platform ecosystems, we hypothesize the effect of the announcement of digital platform strategies on investors’ reactions in the banking industry. We further hypothesize factors that may moderate the relationship between digital platform strategies and stock market returns.

### Main Effect: Digital Platform Strategy Contributing to Stock Market Returns in Banking

As previously outlined, digital platform strategies have emerged in the banking industry in recent years as a response to the increasing success of fintech startups and regulatory requirements.

A digital platform strategy allows incumbent banks to enroll established companies and fintech startups as third-party developers on the digital platform [77]. Banks trigger generativity in the digital platform ecosystem by providing APIs and further resources for developers, creating a potential for digital innovation. Digital innovation contributes to banks’ profitability [88] and growth [5].

Network effects further contribute to the positive impact of digital platform strategies [47]. On the one hand, incumbent banks’ established customer base helps attract initial third-party developers. Thus, the “chicken-and-egg problem,” a crucial challenge during the launch of digital platforms [33], is less critical than for startups. On the other hand, the innovations created on the platform help banks attract and retain customers and find new revenue opportunities [39]. Considering these effects, investors might react positively to the announcement.

While investors might worry about the tensions a digital platform strategy can create within incumbent companies [64, 93], publicly announcing a platform strategy indicates that significant tensions did not occur or were resolved before the announcement. Based on these considerations, we expect that investors will appreciate the announcement of a digital platform strategy. Therefore, we establish the following hypothesis:

Hypothesis 1 (H1): The announcement of a digital platform strategy is positively related to the stock market returns of banks.

### Moderating Effects: Development Status of the Market and AI Orientation

In addition to the main effect of a digital platform strategy on the stock market returns of banks, we hypothesize the impact of *developed market* and *AI orientation* as moderating factors to add nuances to our understanding of the main effect.

#### Developed Market

For two reasons, we included the development status of the banks’ domestic market as a moderating variable. First, the focus of previous event studies on IT-related announcements has been the US (Online Supplemental Appendix 1, Table 1.1). This focus on WEIRD (Western, educated, industrialized, rich, and democratic) contexts might lead to wrong conclusions on the effect of digital platform strategies in other markets, particularly in emerging markets [44, 45, 54]. Including the market’s development status as a moderator allows us to differentiate between developed and emerging markets and provide more nuanced insights into the main effect. Second, the development status of a bank’s domestic market has already been shown to affect the banks’ stock market returns [92]; thus, it should also be considered when evaluating new strategies that banks implement.

Against that backdrop, we argue that banks from developed markets are more likely to benefit from a digital platform strategy because the critical mechanism of platform growth—network effects—has more potential to unfold. In developed markets, more fintech startups have emerged that could act as third-party developers on digital platforms implemented by banks [67]. This environment helps resolve the chicken-and-egg problem when launching a digital platform, that is, the challenge of attracting third-party developers and customers when there are not yet customers for the third-party developers and no offerings for customers [15, 33]. In developed markets, the percentage of the population with a bank account is higher, and they interact more with banks [94]. Similarly, more corporate customers work with banks in developed markets. Thus, the customer side of the digital platform is also more populated, strengthening the network effects on the platform. We assume that investors appreciate this greater potential for network effects, which could significantly grow the banks’ digital platform ecosystems. In sum, we hypothesize:

Hypothesis 2 (H2): For banks from developed markets, the positive effect of a digital platform strategy on stock market returns is stronger than for banks from emerging markets.

#### AI Orientation

We include AI orientation as a moderating factor because AI can support a company’s IT strategy, particularly its digital platform strategy [43].

AI is “the ability of a machine to perform cognitive functions that we associate with human minds, such as perceiving, reasoning, learning, interacting with the environment, problem-solving, decision-making, and even demonstrating creativity” [78, p. iii]. AI builds on machine learning, deep learning, natural language processing, and computer vision [63]. Companies can benefit from using AI because it helps to draw on the ever-growing data with automatic, self-learning algorithms to inform and even take over decision-making [38, 63]. In customer-facing solutions, AI enables companies to interact with customers more efficiently and scalable, for example, by using chatbots in customer service [86, 87]. Companies that put a strategic focus on AI have a high AI orientation; that is, their “overall strategic direction and goals [are] associated with introducing and applying AI technology” [63, p. 1606].

In banking, AI is increasingly used in fraud detection, risk management and cybersecurity, chatbots, algorithmic trading, robo-advisory, credit scoring, asset and wealth management, relationship management, and regulation [46]. We argue that digital platform strategies can benefit from a bank’s AI orientation. First, banks’ AI orientation contributes to generativity in digital platform ecosystems. The scalable data processing systems that banks have to set up in the process of implementing AI technologies can be integrated into the digital platform, supporting third-party developers in implementing AI in their customer-facing apps [63, 71]. For example, U.S. Bank collaborates with IBM to offer AI-based solutions to their customers. It grants third-party developers access to these solutions as part of the bank’s “Banking Innovation APIs” [98].

Second, AI can improve platform processes such as recommendations, improving the quality of the platform offering for customers [1]. Third, customers perceive a platform as having a higher value for them when it has AI capability; that is, when it offers AI-enabled apps and services to them [43]. These effects will strengthen the network effects in the digital platform ecosystem.

In sum, we expect that investors will have a more positive perception of a bank’s digital platform strategy if the bank can leverage the data collected on the platform with AI [43] and offer third-party developers resources to implement AI in their apps and services. We hypothesize:

Hypothesis 3 (H3): For banks that have displayed AI orientation, the positive effect of a digital platform strategy on stock market returns is stronger than for banks that have not shown AI orientation.

## Methodology

We conducted an event study that captured the short-term effect of the announcement of a digital platform strategy on stock market returns [11, 24, 70]. Event studies have been used in management and information systems research to study how announcements regarding, for instance, the impact of decentralization of industries [32], identity theft countermeasures [11], and IT investments [50] impact the stock market returns of companies. Event studies assume that stock prices reflect all publicly available information and that new information is immediately incorporated into stock prices [34, 50]. Hence, observing stock prices and their movements during an announcement allows us to examine investors’ (aggregated) reactions regarding the specific announcement.

### Data and Variables

#### Digital Platform Strategy

To analyze the relationships between the announcement of a digital platform strategy and stock market returns, we first had to identify relevant events, that is, announcements of banks that indicated the launch of a digital platform strategy. In the first step, we compiled a sample of open banking APIs globally from press releases, press conferences, ad hoc disclosures, and articles from open banking or fintech-related news. In this step, we identified over 1,000 companies associated with these APIs. In the second step, because we focus on incumbent banks, we excluded fintech companies, payment providers, and other financial institutions that do not have a banking license. This step left us with 452 banks in our sample. Since our goal was to examine whether the announcement of a digital platform strategy influences a bank’s stock market returns, in the third step, we manually identified whether the banks were publicly listed and whether stock market data was available via the Bloomberg database. This step reduced our sample to 181 publicly listed banks.

In the fourth step, to assign a date to the announcement of the digital platform strategy, we searched all the bank websites to find official press releases about their open banking platform. We enhanced the search of the bank websites by an online search for news articles on each company’s open banking platform. In the case of several announcements made by the same bank, we used the first announcement as the date for the event because we were interested in the effect of the launch of a digital platform strategy.

Once we had an event date for the 181 banks, in the fifth step, we followed the standard practice of excluding possible confounding events or announcements that occur within three days before or after the event to ensure that a bank’s valuation following an announcement is not confounded by these events [70]. All earnings announcements, stock splits, and dividends were considered confounding events. As we used the announcement of a customer-facing AI app or service as an indicator of the AI orientation of the companies (see below for details), we also considered these announcements confounding events. We excluded 12 companies based on standard confounding events and four based on AI-related announcements within the confounding event window.

**Figure 3.**
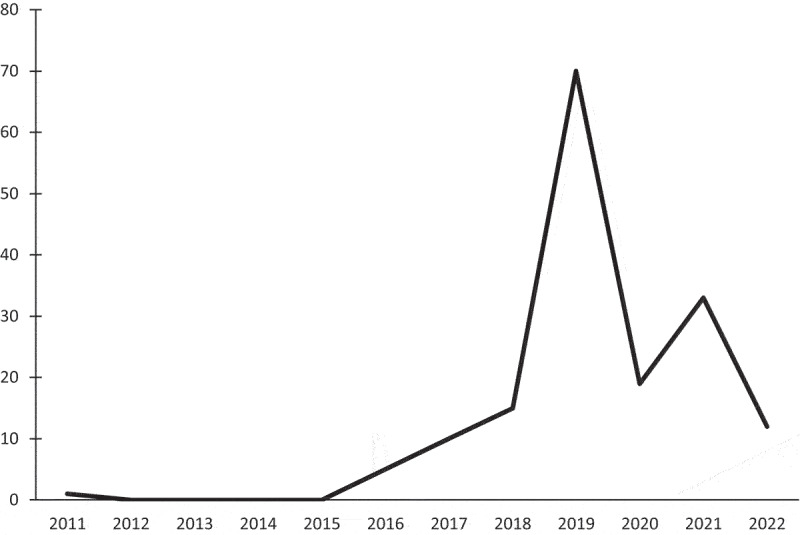
Announcements of digital platform strategies over time.

These steps led to a final sample of 165 banks that had announced a digital platform strategy. Figure 3 illustrates how the events are distributed over time, and Table 1.2 in Online Supplemental Appendix 1 includes examples of the announcements.

Concerning the moderator variables, we classify banks as operating in a *developed market* based on the World Bank’s Country and Lending Groups Classification [95]: We refer to countries as developed markets when they are part of the “High-Income Economies” and as emerging markets when they are part of the “Upper-Middle-Income Economies” or “Lower-Middle-Income Economies.”^2^ The banks in our sample are located in 41 different countries (see Table 1.3 in Online Supplemental Appendix 1). 124 banks are located in developed markets such as the US, UK, and Germany, and 41 are in emerging markets such as India, Brazil, and Indonesia.

We use *AI orientation* to denote whether a bank has displayed AI orientation before announcing its digital platform strategy. To identify AI orientation, we manually searched press releases and news articles for all banks in our sample, assuming that media coverage is a good indicator of companies’ strategic decisions [63, 104]. We used keywords related to customer-facing AI apps and services, including chatbots, AI-based mobile banking apps, virtual advisors, virtual assistants, and robo-advisors. 67 banks in our sample released news related to such AI apps before announcing the digital platform strategy. This step left 98 banks without AI orientation before announcing a digital platform strategy.

### Event Study Analysis

We employ the event study method to test hypothesis H1, that is, to analyze the main effect of whether the announcement of a digital platform strategy leads to higher stock market returns for banks [70]. A detailed description of our approach can be found in Online Supplemental Appendix 2. We drew on the Bloomberg database to obtain the banks’ daily stock prices. Using these stock prices, we calculated daily returns and employed the capital asset pricing model (CAPM) to calculate the banks’ abnormal returns [11, 16].

In line with event windows applied in prior studies [48, 49, 70], for our main analysis, we measure cumulative average abnormal returns over the following event windows, measured in days around the date of the event: [0,1], [0,2], [0,3], [-1,1]; [-2,2], and [-3,3]. For each event window, we applied several parametric tests to evaluate whether the cumulative average abnormal returns during the announcements of a digital platform strategy were statistically significantly different from zero. Besides the standard *t*-test as a parametric test [65], we also report the Patell test [75, 76], the BMP test that has been suggested by Boehmer et al. [8], and the KP-adjusted version of both tests, following Kolari and Pynnonen [59]. We use these alternative parametric tests because the *t*-test has limitations concerning event-induced volatility and cross-sectional correlation of abnormal returns.

For the subsample of banks from developed markets, we also employ the Fama-French three-factor model (FFM) [35] instead of the capital asset pricing model. The FFM has been shown to capture market risks better [11, 35]; however, the required correction factors (size correction factor (SML) and book-to-market correction factor (HML)) are only available for developed markets on a daily basis [36]. We thus only report results based on the FFM as part of the analysis of market development status as the moderating effect. Details on the implementation of the event study using the FFM are provided in Online Supplemental Appendix 2.

## Results

We first report on the findings of the main effect, that is, the effect of digital platform strategy announcements on the stock market returns of banks. Then, we analyzed the effect of two moderators: *developed market* and *AI orientation*.

### Main Effect of Digital Platform Strategies

We found the cumulative abnormal average returns associated with the announcement of a digital platform strategy to be positive in all event windows. This effect was significant at the 1 percent level for the [0,2], [0,3], [-2,2], and [-3,3] event windows as evaluated with the *t*-test (Table 3). The other tests all exhibit significance at varying levels for the four event windows, confirming the findings. Findings for the event window [-3,3] are most significant, with all tests showing significance at the 1 percent level.

**Table 3.** Analysis of the main effect.

**Event window**	**[0,1]**	**[0,2]**	**[0,3]**	**[-1,1]**	**[-2,2]**	**[-3,3]**
CAAR (percent)	0.28	0.70	0.75	0.34	0.94	1.37
p-value *t*-test	0.19	**0.01**	**0.01**	0.20	**0.01**	**0.00**
p-value Patell	0.34	**0.03**	**0.04**	0.40	**0.02**	**0.00**
p-value Patell (KP-adjusted)	0.40	**0.05**	**0.07**	0.45	**0.04**	**0.00**
p-value BMP	0.36	**0.04**	**0.05**	0.39	**0.03**	**0.00**
p-value BMP (KP-adjusted)	0.42	**0.07**	**0.08**	0.44	**0.05**	**0.00**
Share of positive CAR (percent)	52.73	59.39	60.00	52.73	60.61	61.82

N = 165. CAAR: cumulative average abnormal returns; KP: Kolari and Pynnonen [59]; CAR: cumulative abnormal returns; BMP: Boehmer, Musumeci, and Poulsen [8]. p-values lower than a 10 percent level of significance are highlighted in bold.

The returns for these event windows ranged from 0.70 percent to 1.37 percent. Thus, cumulated over the event windows, banks that announced a digital platform strategy saw positive abnormal returns of 0.70 percent to 1.37 percent of their stock market value, on average, depending on the timeframe.

As announcements regarding a digital platform strategy do not fall under strict reporting requirements, potential leakages can be expected and require event windows that begin before the announcement [70]. When comparing the effects measured for the event windows [0,2] and [-2,2], we observe that including the two days before the announcement increases the effect. The same can be observed for the event windows [0,3] and [-3,3].

In an additional robustness test (Online Supplemental Appendix 2, Table 2.1), we only examined the days before the announcement: Although the average abnormal returns for the day before the announcement [-1] and the two days before the announcement [-2,-1] are not significant, they are significant when aggregated over the three-day window before the announcement [-3,-1]. This outcome might further suggest that at least some information about the announcement was available to stakeholders shortly before the event date.

We also calculated the percentage of positive cumulative abnormal returns for each event window. The results show that in all event windows, the cumulative abnormal returns are over 50 percent, which means that the stock market returns of banks will be positively affected by the announcement of a digital platform strategy for a majority of the banks. For the event windows that exhibit significantly positive cumulative average abnormal returns, the share of positive cumulative abnormal returns is around 60 percent. In sum, the event study results support H1—that digital platform strategy announcements positively affect stock market returns.

This finding is robust to using local currencies instead of U.S. dollars as the currency for stock prices (Online Supplemental Appendix 2, Table 2.2) and an even broader market index (Online Supplemental Appendix 2, Table 2.3). As the announcement of a digital platform strategy might be more likely by banks with specific characteristics, we also account for a potential selection bias in our data (Online Supplemental Appendix 2, Table 2.4, and Table 2.5).

### Moderating Effects of Developed Status of the Market and AI Orientation

Next, we analyzed the effect of *developed market* and *AI orientation* as moderating factors (Table 4). We can examine both moderating factors separately as their correlation is low, with -0.096 (-0.163) for the Spearman (tetrachoric) correlation.

**Table 4.** Analysis of the moderating effects.

Event window	[0,2]			[0,3]			[-2,2]			[-3,3]		
Panel A: Developed market Yes: N = 124 | No: N = 41	Yes	Yes^a^	No	Yes	Yes^a^	No	Yes	Yes^a^	No	Yes	Yes^a^	No
CAAR (percent)	0.53	0.25	1.19	0.60	0.31	1.21	0.80	0.46	1.35	1.18	0.71	1.98
p-value *t*-test	**0.06**	0.33	**0.06**	**0.07**	0.30	**0.10**	**0.03**	0.16	**0.10**	**0.01**	**0.07**	**0.04**
p-value Patell	**0.10**	0.45	0.11	0.14	0.48	0.14	**0.10**	0.30	**0.10**	**0.02**	**0.09**	**0.05**
p-value Patell (KP-adjusted)	0.16	0.52	0.11	0.21	0.55	0.14	0.15	0.38	**0.10**	**0.04**	0.14	**0.05**
p-value BMP	0.15	0.51	0.11	0.17	0.51	0.11	0.11	0.31	**0.07**	**0.02**	**0.09**	**0.02**
p-value BMP (KP-adjusted)	0.22	0.58	0.11	0.24	0.58	0.11	0.17	0.40	0.08	**0.04**	0.15	0.02
Share of positive CAR (percent)	58.87	57.26	60.98	58.06	58.06	65.85	60.48	55.65	60.98	60.48	60.48	65.85
Panel B: AI orientation												
Yes: N = 67 | No: N = 98	Yes	No		Yes	No		Yes	No		Yes	No	
CAAR (percent)	0.92	0.54		0.89	0.65		1.07	0.84		1.19	1.51	
p-value *t*-test	**0.03**	0.11		**0.07**	**0.09**		**0.05**	**0.06**		**0.07**	**0.00**	
p-value Patell	**0.03**	0.27		0.13	0.18		0.11	0.11		0.16	**0.00**	
p-value Patell (KP-adjusted)	**0.03**	0.33		0.13	0.23		0.11	0.15		0.16	**0.01**	
p-value BMP	**0.09**	0.26		0.20	0.15		0.15	**0.09**		0.19	**0.00**	
p-value BMP (KP-adjusted)	**0.09**	0.32		0.19	0.20		0.14	0.13		0.19	**0.00**	
Share of positive CAR (percent)	64.18	56.12		65.67	56.12		62.69	59.18		62.69	61.22	

^a^Using the Fama-French model [35] instead of the capital asset pricing model. CAAR: cumulative average abnormal returns; BMP: Boehmer, Musumeci, and Poulsen [8]; KP: Kolari and Pynnonen [59]; CAR: cumulative abnormal returns. p-values lower than a 10 percent level of significance are highlighted in bold.

Like Bose and Leung [11], we compare the cumulative abnormal average returns of the subsamples based on the classifications of *developed market* and *AI orientation*. We thereby focus on the event windows [0,2], [0,3], [-2,2], and [-3,3] because they take into account the time needed for the information to reach investors and, for the two latter, potential leakage. These event windows also exhibited significant results in the main analysis.

First, we compared the subsamples of banks from developed and emerging markets (see Panel A in Table 4). Interestingly, the cumulative average abnormal returns are consistently higher for banks from emerging markets in all four event windows. This observation holds, regardless of whether the results for banks for developed markets are based on the CAPM or the FFM.

Considering the CAPM results, the *t*-test is statistically significant for banks from developed and emerging markets, at least at the 10 percent level for all event windows. For the event windows [-2,2] and [-3,3], most of the other tests also show significant results, and for the event window [-3,3], the findings are supported by the results from the FFM. Across all event windows, the percentage of positive cumulative abnormal returns is higher for banks from emerging markets than those from developed markets, regardless of whether we consider the CAPM or the FFM.

Therefore, our findings are contradictory to H2, which states that the effect of a digital platform announcement on firm value would be stronger for banks from developed markets. We thus reject H2.

Second, we analyze the role of AI orientation and whether banks displayed AI orientation before announcing the digital platform strategy (see Panel B in Table 4).^3^ In line with H3, we see higher cumulative average abnormal returns for banks with AI orientation in the event windows [0,2], [0,3], and [-2,2]. Except for banks without AI orientation in the window [0,2], at least the *t*-test shows significant results. However, in the event window [-3,3], the cumulative average abnormal returns are higher for banks without AI orientation, and for this subsample, all tests show highly significant results. The share of positive cumulative abnormal returns is consistently higher for banks with AI orientation for all four event windows; however, the differences are negligible for the window [-3,3]. Overall, we can only partially confirm H3.

To jointly test for statistical differences between the subsamples, we apply a cross-sectional regression as recommended by MacKinlay [65] and used in previous event studies [3, 10, 21] (Online Supplemental Appendix 2, Table 2.7). The results from this robustness test provide some evidence that AI orientation has a significant moderating effect in the event window [0,2]. In addition, we applied a propensity score matching before the cross-sectional regression to further reduce the risk of estimation biases that arise from bank characteristics (Online Supplemental Appendix 2, Table 2.7). Although the matched sample reduces the sample size, we find evidence that the developed market characteristic has a significant negative moderating effect in the event window [-3,3] and AI orientation in the event window [0,2].

### Post Hoc Analysis: Interaction of Developed Market and AI Orientation

The subsample analysis showed that AI orientation contributes to the effect of a digital platform strategy on stock market returns in three of the four event windows. However, the difference in the cumulative average abnormal returns between banks with and without AI orientation is relatively low and not consistent across all four event windows. To explore this further, we analyzed the sub-subsamples of *developed market* and *AI orientation* as part of a post hoc analysis (Table 5).

**Table 5.** Post hoc analysis of the sub-subsamples of developed market and AI orientation.

Event window	[0,2]		[0,3]		[-2,2]		[-3,3]	
Panel A: Developed market N = 124	AI N = 47	Non-AI N = 77	AI N = 47	Non-AI N = 77	AI N = 47	Non-AI N = 77	AI N = 47	Non-AI N = 77
CAAR (percent)	0.60	0.48	0.45	0.67	0.65	0.88	0.52	1.57
p-value *t*-test	0.21	0.16	0.41	**0.09**	0.30	**0.05**	0.49	**0.00**
p-value Patell	**0.06**	0.53	0.30	0.29	0.26	0.22	0.47	**0.01**
p-value Patell (KP-adjusted)	**0.06**	0.59	0.29	0.36	0.25	0.29	0.46	**0.03**
p-value BMP	0.16	0.54	0.41	0.27	0.35	0.19	0.54	**0.00**
p-value BMP (KP-adjusted)	0.15	0.60	0.40	0.35	0.34	0.27	0.53	**0.01**
Share of positive CAR (percent)	65.96	54.55	63.83	54.55	63.83	58.44	59.57	61.04
Panel B: Emerging market N = 41	AI N = 20	Non-AI N = 21	AI N = 20	Non-AI N = 21	AI N = 20	Non-AI N = 21	AI N = 20	Non-AI N = 21
CAAR (percent)	1.70	0.74	1.95	0.53	2.11	0.67	2.78	1.24
p-value *t*-test	**0.05**	0.42	**0.05**	0.62	**0.06**	0.58	**0.04**	0.39
p-value Patell	0.27	0.24	0.22	0.38	0.23	0.26	0.14	0.20
p-value Patell (KP-adjusted)	0.26	0.22	0.21	0.36	0.22	0.25	0.14	0.18
p-value BMP	0.35	0.16	0.24	0.28	0.14	0.27	**0.08**	0.11
p-value BMP (KP-adjusted)	0.35	0.15	0.24	0.26	0.14	0.25	**0.08**	**0.10**
Share of positive CAR (percent)	60.00	61.90	70.00	61.90	60.00	61.90	70.00	61.90

CAAR: cumulative average; BMP: Boehmer, Musumeci and Poulsen [8] abnormal returns; KP: Kolari and Pynnonen [59]; CAR: cumulative abnormal returns; AI: AI orientation. p-values lower than a 10 percent level of significance are highlighted in bold.

The findings of the post hoc analysis show that the inconsistent impact of AI orientation comes from the subsample of banks from developed countries (see Panel A in Table 5). Even though the findings are less significant—potentially due to the smaller sizes of the sub-subsamples—we interpret that for the panel of banks from developed markets, banks without AI orientation see higher cumulative average abnormal returns for the event windows [0,2], [0,3], and [-2,2]. Similar to the subsample analysis, this effect changes direction in the event window [-3,3], giving no clear indication of whether AI orientation displayed before the announcement of a digital platform strategy is beneficial for banks from developed markets.

For example, the Dutch bank ING introduced customer-facing AI solutions in 2017 [51] and announced its digital platform strategy in 2018 [52]. However, despite its prior AI orientation that could contribute to the generative effect of its digital platform, ING saw negative abnormal returns after announcing its digital platform strategy across all event windows.

For banks from emerging markets—which already see higher cumulative average abnormal returns than banks from developed markets—AI orientation seems to increase this effect further (see Panel B in Table 5). For all event windows, cumulative average abnormal returns are higher for banks with AI orientation, and the *t*-test shows significance across all windows. The percentage of positive cumulative abnormal returns is not always higher for banks with AI orientation; thus, relatively few banks, seen as innovative and promising by investors, drive the effect.

One example includes the Thai bank Krungsri (Bank of Ayudhya PCL), which launched an Open API platform in 2021 [81]. The bank had already rolled out customer-facing AI apps since 2017, for example, by including robo-advising features in its mobile banking app [4].

## Discussion

In this section, we first summarize and interpret our findings in view of the literature on digital platforms, suggesting avenues for future research. Then, we outline our study’s contributions to practice and limitations.

### Digital Platform Strategies as Opportunities for Incumbents

Our findings confirmed the positive main effect of digital platform strategies on the stock market returns of banks, in line with Hypothesis 1. Investors seem to believe the potential benefits of digital platform strategies outweigh the challenges of implementing them.

The mechanisms that have driven the growth of digital platforms in the IT industry—generativity [18, 105] and network effects [12, 47, 55, 73]—can also help incumbent banks to enable innovation in their digital platform ecosystem and grow their customer base. For example, the Australian airline Qantas used Citi’s digital platform to create an app that lets Qantas frequent travelers manage their Qantas rewards along with their Qantas credit cards while also providing an overview of their bank accounts with Citi. Such an offering can attract new customers for Citi who use Qantas credit cards and switch their bank accounts to Citi because of the convenient integration with Qantas products.

These results on the main effect and their interpretation contribute to information systems literature on digital platforms. To the best of our knowledge, this study is the first to quantitatively measure the impact of digital platform strategies on the investors’ perception of companies in traditional industries. The indications for a positive effect add to the limited and mixed evidence on digital platform strategies implemented by incumbents [82, 93, 102]. Thus, while incumbents must overcome the challenges identified in the previous literature, investors will likely appreciate these efforts.

While we focused on the banking industry as the context for our study, we argue that our findings are also relevant for incumbent companies from other traditional industries. On an abstract level, a digital platform strategy’s potential benefits and challenges are similar across traditional industries. Generativity and network effects allow growth, while tensions between internal and external collaboration must be resolved [93]. In industries such as insurance, health care, manufacturing, and even agriculture, there is great potential for digital innovation, and our findings indicate that investors might appreciate it if incumbents strive to leverage this potential. In addition, the banking industry is more advanced than other traditional industries concerning the adoption of digital platform strategies. It can serve as a trailblazer industry, inspiring other industries to follow.

### Digital Platform Strategies in Emerging Markets

We did not confirm the hypothesized positive moderation effect of companies located in developed markets (H2). Contrarily, we found that investors reacted more positively to banks from emerging markets announcing a digital platform strategy.

This finding shows that digitalization approaches, such as digital platform strategies, might pay off for banks in emerging markets even more than for banks in developed markets. One explanation could be the growth potential of financial services in emerging markets that is, amongst others, driven by the high adoption rates of mobile banking [29, 68, 99]. For example, M-Pesa, a phone-based payment service that originated in Kenya [69], reached 52.4 million users in 2022 [100]. With a digital platform strategy, banks might be able to quickly roll out more digital financial services to users of mobile banking. This effect might also help banks overcome the chicken-and-egg problem when they launch a digital platform and strengthen network effects beyond the initial launch.

Future research could evaluate which banks in emerging markets see strong effects and identify reasons why other banks do not benefit. Scholars could explore differences between emerging markets and between banks in qualitative case studies on digital platform strategies that have primarily been conducted in WEIRD domains [58, 82, 84, 93]. We thus support the broader call for more information systems research in GREAT (Growing, rural, Eastern, aspirational, and transitional) domains [54].

In addition to qualitative work, better data for emerging markets could enhance our understanding of digital platform strategies in the banking industry in emerging markets. For example, the availability of daily instead of monthly correction factors would allow us to replace the CAPM market model with the more sophisticated Fama-French three-factor model as a basis for the event study [37]. Thus, the current focus on developed markets in research on digital platforms could be overcome, leading to a more nuanced understanding of the potential of digital platforms [54, 55].

### Digital Platform Strategies and Their Interplay with AI Orientation

We found evidence that AI orientation contributes positively to the investors’ reaction to the announcement of digital platform strategies for some of the event windows we studied. Therefore, we only partially confirmed our third hypothesis. However, a post hoc analysis suggests that banks from emerging markets see a robust moderating effect of AI orientation. In contrast, banks from developed markets see only a small or no such effect.

We argued that AI orientation could help incumbent banks to increase the generativity of their digital platforms by providing third-party developers access to AI technology and that AI-powered apps for customers can further strengthen network effects. In developed markets, several considerations of investors could be at play, leading to inconsistent reactions. First, banks that have already announced and, in many cases, implemented customer-facing AI solutions have displayed AI orientation and the IT capabilities required for innovative approaches to leveraging IT. When they announce a digital platform strategy, investors who have already factored in the companies’ IT capabilities might see this as consequential rather than surprising.

Second, for banks that have already announced customer-facing AI solutions, investors might see it as more promising for them to join existing digital platform ecosystems of other banks or consortia rather than launching their own digital platform. Such a “belong” strategy can be more beneficial than a “build” strategy because it avoids the chicken-and-egg problem during the platform launch and allows the company to reach customers directly through the ecosystem that the bank would join [25]. For example, suppose a bank has launched a successful robo-advisory service. In that case, it might benefit from making it available in various ecosystems rather than offering the technology behind it to third-party developers on their own platform.

Third, investors might be concerned about banks’ AI orientation combined with a digital platform strategy. On the one hand, investors might be skeptical about the impact of AI orientation because incumbents often struggle with their cumbersome legacy systems and inflexible organizational processes when implementing AI solutions [56]. In this case, investors would react less positively to the announcement of a digital platform strategy because for the platform strategy to be successful, the bank needs a well-orchestrated stack of core technologies. On the other hand, when a bank launches customer-facing AI solutions, IT increasingly takes over decisions on how to best support and consult customers [60]. A digital platform strategy that grants generativity to third-party developers while offering such customer-facing AI solutions might be perceived as risky because that combination reinforces issues of trust, privacy, and security [6, 20]. The more data, especially customer data, is accumulated on the platform, the more severe the consequences of misuse.

In contrast, in emerging markets, investors might appreciate the AI orientation of incumbent banks more because it might help banks grow their platforms faster. In particular, the predominant mobile use of banking solutions allows incumbent banks to benefit from innovative AI-powered apps in their ecosystem. Thus, the announcement of a digital platform strategy when the banks have already displayed AI orientation could be interpreted by investors as a signal that these banks are “leapfrogging” into digital banking [22].

In addition, investors might value the risks of combining AI solutions and open banking differently than in developed markets because most cyber attacks target companies in developed markets [62]. Thus, in the investors’ view, incumbent banks in emerging markets have the most significant potential to benefit from a combined strategy of AI and digital platforms.

Our results unearthed the complex interplay between AI orientation and digital platform strategies. Future research could therefore shed more light on how AI is implemented, for example, by considering the role of AI in the banks’ operating models, core technology, decision-making, and engagement [7]. Then, future research could analyze the cognitive reapportionment [60] that is unfolding in the financial industry as the use of AI spreads and what implications that has for digital platform strategies. Banks might need new types of digital platforms that have been referred to as cognitive platforms [60], digital platforms designed for human-AI hybrids [78], and AI-based platforms [1] to leverage the synergies between the technologies. For example, future work could implement event studies in which the announcements of customer-facing AI solutions represent the events. Qualitative case studies could focus on the temporal aspect of which strategic move should come first in which situations.

### Contribution to Practice

Our findings contribute to practice. First, we highlight that digital platform strategies are not just an often-hyped topic but can lead to higher stock market returns of companies, indicating a positive effect on performance. Companies from traditional industries, such as the banking industry, might benefit from going beyond just granting access to customer and transaction data by implementing a full-scale platform strategy that fuels generativity and network effects in an emerging digital platform ecosystem.

Second, given that we used the announcement of digital platform strategies as the independent variable, our findings also show that just announcing a digital platform strategy might positively affect stock market returns, even if the strategy is delayed or not implemented. While this is a limitation of our study, companies could exploit this so-called “announcement effect” [31] to increase a firm’s stock market returns in the short run, for example, before an acquisition. Concerning the banks in our sample, we did not observe examples of banks that announced a digital platform strategy without actually implementing it.

Third, to the best of our knowledge, our study is the first to provide quantitative results on the potential of digital platform strategies in emerging markets. Based on our findings, established companies in emerging markets from the banking industry and beyond should consider digital platform strategies, ideally along with implementing an AI strategy.

### Limitations

This study is subject to several limitations that yield questions for future research. First, we looked at two differences between the banks in our sample to run subsample analyses (the development status of their domestic market and their AI orientation). Further factors might impact whether a digital platform strategy leads to higher stock market returns. For example, the top management team’s digital expertise and whether investors trust these capabilities might have a moderating effect. More nuanced insights might be gained by including data on the “digital upper echelon” [41] of firms implementing digital platform strategies. Furthermore, digital platform strategies might differ in their characteristics [2]. For example, the amount and quality of resources offered to third-party developers on the platform could vary, impacting the quality of apps and services created [101]. We argue that this difference is not yet observable for investors at the time of the announcement, but future studies could investigate the implications of varying platform quality on generativity.

Second, our study analyzed the impact of the announcement of a digital platform strategy on stock market returns. Thus, our findings do not indicate whether implementing the digital platform strategy will succeed. The success might also depend on the governance of the digital platform ecosystem [82]. Future work could study the long-term impact of digital platform strategies, for example, with a calendar portfolio analysis [61]. Alternatively, future analyses could use other dependent variables, such as Tobin’s Q in cross-sectional regressions, and the results could be compared to the effects identified in an event study [11].

Third, open banking regulations such as the EU Payment Services Directive regulation (PSD2) and the UK Open Banking Standard [72] might also have impacted the implementation of digital platform strategies. We did not focus on the role of regulation because we think that the investors’ reaction captures the growth potential of the digital platform, regardless of whether regulatory requirements played a role in the decision to implement it. Future work could specifically study the impact of regulation by comparing markets with and without open banking regulation.

## Conclusion

In this study, we analyzed whether the digital platform strategies of companies are perceived positively by investors, as measured by short-term stock market return. Using the banking industry as a context, we identified a positive effect of a digital platform strategy on a bank’s stock market returns. This effect is stronger for banks from emerging markets, illustrating the growth potential of banks in emerging markets and the value of digital platforms to leverage this potential. The interplay between AI orientation and digital platform strategies is more ambiguous, which calls for more research on the role of AI-based or cognitive platforms in the banking industry.

## Supplementary Material

Supplemental Material

## Notes

^1.^
We thank one of the anonymous reviewers for pointing this out.

^2.^
We thank one of the anonymous reviewers for this comment. No bank in our sample was part of the “Low-Income Economies.”

^3.^
In an additional analysis, we analyzed the opposite order of strategy implementation, that is, the display of AI orientation at a point in time when a bank has already implemented a digital platform. This analysis shows a similar positive effect of the display of AI orientation on the investors’ perception, indicating that a digital platform strategy and AI orientation have a mutually reinforcing interplay. We consider this interplay as an issue for future research and thank one of the anonymous reviewers for pointing out these two strategic scenarios.
