# Supplementary material for: The Effect of Digital Platform Strategies on Firm Value in the Banking Industry
Source: J Manag Inf Syst. 2024 Jun 24;41(2):394–421. doi: 10.1080/07421222.2024.2340825 (PMC11225948; doi:10.1080/07421222.2024.2340825)
Supplement: Supplemental Material [file MMIS_A_2340825_SM0212.pdf]

Online Supplemental Appendix—The Effect of Digital Platform Strategies on Firm Value in the Banking Industry

Appendix 1

Previous Event Studies on IT-Related Announcements

Source	Outlet	Year	Definition of IT-related announcement	Positive abnormal returns?	Geographical scope
Dos Santos, Peffers and Mauer [14]	Information Systems Research	1993	*IT investment:* "purchases, agreements to purchase, or plans to buy equipment, software, or services" (p. 5)	No	USA
Im, Dow and Grover [20]	Information Systems Research	2001	*IT investment:* purchase, installation, and development of computer hardware and software	Yes, for smaller firms and IT investments after 1991	USA
Subramani and Walden [31]	Information Systems Research	2001	*E-commerce initiative:* Announcement to conduct business via the Internet	Yes, higher for business-to-consumer initiatives and tangible products	USA
Chatterjee, Pacini and Sambamurthy [10]	Journal of Management Information Systems	2002	*IT infrastructure investment:* investment in "communication network services, messaging services, database management services, security services, disaster planning, and technology support" (p. 17)	Yes, higher for IT infrastructure investments than for IT application investments	USA
Dehning, Richardson and Zmud [13]	MIS Quarterly	2003	*IT investment:* In line with definitions by [10, 14, 20];<br>*Transformative IT investment:* investments that contribute to radical business models that disrupt industry practices and market structures	Yes, for transformative IT investments	USA
Dehning, Richardson, Urbaczewski and Wells [12]	Journal of Management Information Systems	2004	*E-commerce initiative:* in line with [31]	Yes, for announcements in 1998;<br>No, for announcements in 2000	USA
Ranganathan and Brown [30]	Information Systems Research	2006	*Enterprise resource planning (ERP) investments:* adoption of an ERP solution	Yes, for ERP projects with higher functional or physical scope	USA
Agrawal, Kishore and Rao [1]	Information & Management	2006	*E-business outsourcing:* outsourcing of IT tasks and projects	Yes	USA
Meng and Lee [24]	Decision Support Systems	2007	*IT investments:* investments in/purchase of computer hardware and software	Yes, for IT investments in China;<br>No, for IT investments in the USA	USA and China
Chai, Kim and Rao [9]	Decision Support Systems	2011	*Information security investment:* purchase of information security solutions	Yes	USA
Bose and Leung [6]	MIS Quarterly	2019	*Identity theft countermeasures:* adoption of "technical solutions to prevent and detect identity theft, and mitigate their negative impact on firms" (p. 314)	Yes	Global (39 countries)

Table 1.1. Previous Event Studies on IT-Related Announcements

Examples of Announcements Related to Digital Platform Strategies

**Title:**	Abu Dhabi's ADIB launches API developer portal to drive open banking
**Date:**	Jun 14, 2022
**Publication:**	Gulf Business
**Excerpt:**	Abu Dhabi Islamic Bank (ADIB) has launched its first application programming interface (API) developer portal. The new ADIB API developer portal will drive open banking in the market, allowing fintech developers to build new products that interact with the lender's platforms.
**Title:**	AIB opens APIs in Ireland
**Date:**	Nov 02, 2018
**Publication:**	OpenBanking Expo
**Excerpt:**	AIB has launched Ireland's first industry standard* Open Application Programming Interfaces (APIs). This technology now allows customers to benefit from secure trusted technology from third-party providers (TPPs), once they give their permission to do so. Open APIs are a proven way to allow trusted TPPs to access, should the customer choose, their financial data in order to offer the customer a banking service directly, meaning innovative new solutions can be developed by external partners. AIB was one of the first banks to launch APIs in the UK earlier this year and is now adopting the same standard in Ireland.
**Title:**	Take advantage of the new opportunities available via the Alpha Bank Group API Portal!
**Date:**	May 24, 2019
**Publication:**	Alpha Bank
**Excerpt:**	The aim of the Alpha Bank Group API Portal is to make banking APIs available to programmers, start-ups, and in general to all those with an interest in innovation. Interested parties can complete their registration online on the Alpha Bank Group API Portal, obtain information on the available APIs and familiarize themselves with them in the Sandbox test environment. Alpha Bank Group API Portal has been in production since 24.05.2019.
**Title:**	Banca IFIS tests Open Banking with API technology
**Date:**	Mar 14, 2019
**Publication:**	Banca Ifis
**Excerpt:**	Banca IFIS embraces the European Directive on Open Banking and PSD2 payments, ready to share its data with the various players in the banking ecosystem. As from 14 March 2019, Banca IFIS, as a payment service provider with accessible online accounts, will provide a dedicated interface for 'Third Parties 'to connect via API to the banking services offered for the trial.
**Title:**	BBVA launches its Open Banking business
**Date:**	Nov 16, 2017
**Publication:**	BBVA
**Excerpt:**	BBVA is kickstarting the launch of its open banking program by making eight of its APIs commercially available for the first time.

Table 1.2. Examples of Announcements Related to Digital Platform Strategies

**Title:**	Crédit Agricole Is Making A PSD2 API Available to Approved Payment Services Providers
**Date:**	Mar 14, 2019
**Publication:**	Crédit Agricole
**Excerpt:**	In accordance with the second Payment Services Directive (PSD2), the Crédit Agricole regional banks are making their API available to payment services providers (PSPs), in order to comply with PSD2's Regulatory Technical Standard (RTS).
**Title:**	Reimagining banking, DBS launches world's largest banking API developer platform
**Date:**	Nov 02, 2017
**Publication:**	DBS
**Excerpt:**	DBS Bank today set yet another global milestone with the launch of a banking API developer platform that is the largest by a bank anywhere in the world.
**Title:**	ING launches Developer Portal
**Date:**	May 24, 2018
**Publication:**	ING
**Excerpt:**	ING has opened a Developer Portal to accelerate innovation by working more closely with external developers. This first version of the portal gives developers access to selected, simulated ING application programming interfaces (APIs) they can use to create new and innovative customer experiences jointly.
**Title:**	The APIs of KBC Bank, KBC Brussels and CBC Banque are available
**Date:**	Feb 26, 2019
**Publication:**	KBC
**Excerpt:**	KBC/CBC's APIs are ready - As a progressive player in the financial sector and one of the pioneers of PSD2, we have made an extra effort to ensure that our APIs become available as quickly as possible. The APIs of KBC Bank, KBC Brussels and CBC Banque are ready for use.
**Title:**	RBC first Canadian bank to open an API developer portal
**Date:**	Mar 20, 2018
**Publication:**	RBC
**Excerpt:**	With a focus on driving innovation to deliver leading-edge solutions for its clients and support the Canadian innovation community, RBC is the first Canadian bank to launch an Application Programming Interface (API) developer portal. The RBC Developers portal will allow eligible, external software developers, industry innovators, and clients to access select RBC APIs.

Table 1.2. Continued

List of Countries of Origin

Developed markets		Emerging markets	
**Country**	**Count**	**Country**	**Count**
USA	22	India	11
Italy	12	Brazil	5
UK	10	Indonesia	5
Denmark	8	South Africa	4
Canada	7	Turkey	3
Spain	4	Thailand	3
Germany	4	Colombia	2
Poland	4	China	2
Finland	4	Pakistan	1
Austria	4	Kenya	1
France	4	Bangladesh	1
Switzerland	4	Nigeria	1
United Arab Emirates	4	Sri Lanka	1
Australia	3	Philippines	1
Sweden	3		
Ireland	3		
Greece	3		
Norway	3		
Netherlands	2		
Belgium	2		
Portugal	2		
Japan	2		
Hong Kong	2		
Oman	2		
Singapore	1		
Cyprus	1		
Hungary	1		
Lithuania	1		
Estonia	1		
Romania	1		
**Sum**	**124**	**Sum**	**41**

Table 1.3. List of Countries of Origin (classification based on The World Bank's Country and Lending Groups Classification [32]: developed markets are part of the "High-Income Economies," emerging markets are part of the "Higher-Middle-Income Economies" or "Lower-Middle-Income Economies")

Appendix 2

Details on the Event Study Analysis

As we ensured that all banks were available in the Bloomberg database, we used the Bloomberg Excel add-in to obtain the respective stock prices.[1] After calculating returns from the banks' stock prices, we used the market model based on the capital asset pricing model (CAPM) to calculate the banks' abnormal returns [6, 8][2]. For this purpose, we estimated the following Ordinary Least Squares (OLS) model:

$$R_{it} = \alpha_i + \beta_i R_{mt} + \varepsilon_{it} \quad (1)$$

where R_{it} is the return for company i on day t, R_{mt} is the return of the market index m on day t and ε_{it} is the error term. As a market index, we used the MSCI World Index, also obtained from Bloomberg (ticker: MXWO Index), for the main analysis.[3]

To avoid a bias in the parameter estimations around the event date, we estimated the parameters using the market model with a 200-trading-day window ending 20 days before the event day [7]. In the next step, we used the estimated parameters $\hat{\alpha}_i$ and $\hat{\beta}_i$ from the market model in equation (1) and calculated abnormal returns AR_{it} for each bank as follows:

$$AR_{it} = R_{it} - \left(\hat{\alpha}_i + \hat{\beta}_i R_{mt}\right) \quad (2)$$

---

[1] In the main analysis, we opted for obtaining all stock prices in USD via the Bloomberg Excel add-in to calculate returns. In a robustness check, however, we also used local currencies instead of USD (Appendix 2, Table 2.2).
[2] Due to our global and diverse sample of banks and the use of a global market index (see below), we omitted the subtraction of the risk-free rate as there is no global risk-free rate. Technically speaking, we therefore estimate the market model. However, we subtract the risk-free rate when estimating the Fama-French three-factor model for the subsample analysis with banks in developed markets. We thereby followed the approach by Bose & Leung [6].
[3] For robustness, we also used the MSCI ACWI Index (Bloomberg ticker MXWD Index), which includes an even broader range of countries as a market index (Appendix 2, Table 2.3).

We then averaged the individual banks' abnormal returns AR_{it} to receive the average abnormal return AAR_t for each day t:

$$AAR_t = \frac{1}{N}\sum_{i=1}^{N} AR_{it} \tag{3}$$

where N is the number of events (and, in our case, the number of banks). To finally calculate cumulative average abnormal returns ($CAAR[t_1, t_2]$) for a specific event window that ranges from t_1 to t_2, we summed up the respective AAR_t that are included in the event window:

$$CAAR[t_1, t_2] = \sum_{t=t_1}^{t_2} AAR_t \tag{4}$$

We measured cumulative average abnormal returns over the event windows [0,1], [0,2], [0,3], [-1,1]; [-2,2], and [-3,3]. For all analyses, we used Stata's estudy package [27]. The package includes the calculation of cumulative average abnormal returns and the corresponding test statistics. We report the *t*-test [22], the Patell test [28, 29], the BMP test [4], and the KP-adjusted version of both tests, following Kolari and Pynnonen [21]. We report additional parametric tests beyond the *t*-test to address limitations of the *t*-test related to event-induced volatility and cross-sectional correlation of abnormal returns.

The basic idea of parametric tests is to derive a test statistic that follows a specific distribution (e.g., standard normal distribution in the case of the *t*-test when N is large). Implementing such a test typically requires the standard deviation of the cumulative average abnormal return ($\sigma_{CAAR[t_1,t_2]}$) calculated as

$$\sigma_{CAAR[t_1,t_2]} = \frac{1}{N^2}\sum_{i=1}^{N} \sigma^2_{AR[t_1,t_2]} \tag{5}$$

However, as $\sigma^2_{AR[t_1,t_2]}$ is unknown, it is appropriate to use an estimate based on the estimation window $[T_1, T_2]$ where, as outlined above, $T_1 = -220$ and $T_2 = -20$ [22]. Hence, we calculated the standard deviation of the abnormal return using the daily abnormal returns from the estimation window ($\sigma^2_{AR[T_1,T_2]}$) to obtain $\hat{\sigma}^2_{AR[t_1,t_2]}$. For event windows longer than one day, we have to scale $\hat{\sigma}^2_{AR[t_1,t_2]}$ by multiplying it by length of the event window. For the *t*-test, for instance, the test statistic is then derived by

$$test\ statistic = \frac{CAAR[t_1, t_2]}{\sigma_{CAAR[t_1,t_2]}} \tag{6}$$

where the calculation of $\sigma_{CAAR[t_1,t_2]}$ is depicted in equation (5) above. This test statistic will asymptotically follow a standard normal distribution when N is large so we can easily derive its statistical significance. For details on the test statistics of the Patell test [28, 29], the BMP test [4], and their adjusted versions [21], we refer to the respective original sources.

As an alternative to CAPM for calculating abnormal returns, the Fama-French three-factor model (FFM) has been suggested [15] because it captures market risks better. We were unable to use the Fama-French three-factor model for our event study because the required correction factors (size correction factor (SML) and book-to-market correction factor (HML)) were only available for developed markets on a daily basis [16]. However, we used the Fama-French model for the subsample of companies from developed markets as a robustness test for the results obtained based on the CAPM (see Table 4 in the main article). For this purpose, we extend equation (1) above by two factors and estimate the following OLS model:

$$R_{it} - R_{ft} = \alpha_i + \beta_i\left(R_{mt} - R_{ft}\right) + \gamma_i SMB_t + \delta_i HML_t + \varepsilon_{it} \tag{7}$$

where R_{it} is the return for company i on day t, R_{ft} is the risk-free rate of the turn (US treasury bills) on day t, R_{mt} is the return of the market index m on day t, SMB_t is the size correction factor for day t, HML_t is the book-to-market correction factor for day t, and ε_{it} is the error term. The factors for the Fama-French model were obtained from Fama and French [16]. Parameter estimation was again done with a 200-trading-day window ending 20 days before the event day to avoid any bias caused by the events themselves [7].

Then, the abnormal returns AR_{it} were calculated using equation (8), as shown below. Taking the parameters $\hat{\alpha}_i$, $\hat{\beta}_i$, $\hat{\gamma}_i$, and $\hat{\delta}_i$ from the results of the regression based on equation (7).

$$AR_{it} = R_{it} - R_{ft} - \left[\hat{\alpha}_i + \hat{\beta}_i\left(R_{mt} - R_{ft}\right) + \hat{\gamma}_i SMB_t + \hat{\delta}_i HML_t\right] \quad (8)$$

Next, we calculated cumulative average abnormal returns (CAAR) in the same way as in our main analysis, as shown in equations (3) and (4). Based on the CAAR values, we performed the same battery of parametric tests using Stata's estudy package [27]. The results are included in Table 4 in the main article, which shows the analysis of the moderating effects.

Robustness Test – Event Windows before Announcement

**Event window**	**[-1]**	**[-2,-1]**	**[-3,-1]**
CAAR (%)	0.06	0.24	0.63
p-value *t*-test	0.70	0.27	**0.02**
p-value Patell	0.95	0.41	**0.02**
p-value Patell (KP-adjusted)	0.96	0.46	**0.04**
p-value BMP	0.95	0.35	**0.01**
p-value BMP (KP-adjusted)	0.95	0.40	**0.02**
Share of positive CAR (%)	52.73	50.30	60.61

N = 165
CAAR = cumulative average abnormal returns
KP = Kolari and Pynnonen [21]
CAR = cumulative abnormal returns
BMP = Boehmer, Musumeci and Poulsen [4]

Table 2.1. Analysis of the Main Effect for Event Windows Before the Announcement

Robustness Test – Local Currencies

**Event window**	**[0,1]**	**[0,2]**	**[0,3]**	**[-1,1]**	**[-2,2]**	**[-3,3]**
CAAR (%)	0.31	0.67	0.70	0.38	0.90	1.31
p-value *t*-test	0.13	**0.01**	**0.02**	0.14	**0.01**	**0.00**
p-value Patell	0.29	**0.04**	**0.08**	0.33	**0.03**	**0.00**
p-value Patell (KP-adjusted)	0.34	**0.06**	**0.08**	0.38	**0.05**	**0.01**
p-value BMP	0.33	**0.07**	**0.09**	0.32	**0.03**	**0.00**
p-value BMP (KP-adjusted)	0.38	**0.10**	0.13	0.38	**0.05**	**0.00**
Share of positive CAR (%)	52.73	57.58	58.79	52.73	61.21	65.45

N = 165
CAAR = cumulative average abnormal returns
KP = Kolari and Pynnonen [21]
CAR = cumulative abnormal returns
BMP = Boehmer, Musumeci and Poulsen [4]

Table 2.2. Analysis of the Main Effect with Stock Prices in Local Currencies

Robustness Test – Alternative Market Index

**Event window**	**[0,1]**	**[0,2]**	**[0,3]**	**[-1,1]**	**[-2,2]**	**[-3,3]**
CAAR (%)	0.25	0.66	0.71	0.32	0.90	1.33
p-value *t*-test	0.23	**0.01**	**0.02**	0.21	**0.01**	**0.00**
p-value Patell	0.42	**0.04**	**0.06**	0.45	**0.03**	**0.00**
p-value Patell (KP-adjusted)	0.47	**0.06**	**0.09**	0.50	**0.05**	**0.01**
p-value BMP	0.44	**0.06**	**0.08**	0.45	**0.03**	**0.00**
p-value BMP (KP-adjusted)	0.49	**0.10**	0.11	0.50	**0.06**	**0.01**
Share of positive CAR (%)	52.73	59.39	59.39	52.12	60.61	61.21

N = 165
CAAR = cumulative average abnormal returns
KP = Kolari and Pynnonen [21]
CAR = cumulative abnormal returns
BMP = Boehmer, Musumeci and Poulsen [4]

Table 2.3. Analysis of the Main Effect with the MSCI ACWI Index as the Market Index

Robustness Test – Self-Selection Bias

As a digital platform strategy announcement may not be random but determined by specific bank characteristics, our findings might suffer from a potential self-selection bias. To control for this potential bias, we apply—in line with existing studies [6, 11, 23]—a Heckman correction [19].

In the first stage of the Heckman correction, we model the probability of a bank announcing a digital platform strategy. For this purpose, we include the share of other banks in the sample that have already announced a digital platform strategy because it is likely that similar activities from peers affect the focal bank's decision; we refer to this as peer effect. While Wolfolds and Siegel [35] emphasize the careful use of instruments in the Heckman correction, similar identification strategies have been applied by Fotheringham and Wiles [17] in the context of chatbot launches and by Bhagwat, Warren, Beck and Watson [3] in the context of corporate activism. Importantly, such a peer variable satisfies the exclusion restriction as it only has an effect on the probability of the firm's action (i.e., announcing a digital platform strategy) but not on the investors' evaluation of a specific announcement (i.e., abnormal returns after announcing a digital platform strategy).

We furthermore expect large banks to be more likely to implement a digital platform strategy. Therefore, we included total assets as a proxy for size as an independent variable in the first stage of the Heckman correction. To capture all missing variables related to the specific year of implementation, we included year-fixed effects and consequently estimated the following probit model:

$$\begin{aligned}&\text{Probability}(DigitalPlatform_{it} = 1)\\&\qquad = \alpha + \beta_1 \ln(TotalAssets_{it-1}) + \beta_2 PeerEffect_{st} + \gamma_t + \epsilon_{it}\end{aligned} \tag{9}$$

where Probability($DigitalPlatform_{it} = 1$) is a binary variable, being one if bank i has announced a digital platform strategy in year t and zero in the years before. In line with similar studies [e.g., 17], we use the log-transformed total assets of bank i from the previous year (i.e., $t - 1$). As we expect differences between banks operating in developed markets and banks operating in emerging markets, we calculate peer effects depending on the market development status s. Thus, $PeerEffect_{st}$ is defined as the percentage of banks in the developed (emerging) sample that have announced a digital platform strategy in year t. Finally, ϵ_{it} represents the error term.

After estimating equation (9), we calculated the inverse Mill's ratio λ_{it} for every bank i in year t as follows:

$$\lambda_{it} = \phi(\alpha + \beta X)/\Phi(\alpha + \beta X) \tag{10}$$

where X represents a vector that includes the independent variables of equation (9) above. $\phi(\cdot)$ denotes the standard normal probability density function and $\Phi(\cdot)$ the corresponding cumulative distribution function.

For the second stage of the Heckman correction, the inverse Mill's ratio serves as an independent variable to explain the cumulative abnormal returns of the event. Hence, we estimate a simple cross-sectional regression as there is only one observation in the event year for each bank i. Following previous studies [e.g., 5], this cross-sectional analysis uses a standard OLS regression with cumulative standardized abnormal returns ($CSAR_i$) as the dependent variable. More specifically, we estimate the following OLS regression model:

$$CSAR_i = \alpha + \beta_1 \lambda_i + \beta_2 Developed_i + \beta_3 AIorientation_i + \beta_4 EventYear + \varepsilon_i \tag{11}$$

where $CSAR_{it}$ is the cumulative standardized abnormal return for bank i as calculated in, for example, Bose and Leung (2019). λ_i represents the inverse Mill's ratio for every bank i of the event year. Notably, a statistically significant coefficient for λ_i would indicate a potential self-selection bias. $Developed_i$ is a dummy variable being one if bank i is in the *developed market* subsample and zero otherwise. $AIorientation_i$ is a dummy being one if bank i is in the *AI orientation* subsample and zero otherwise. We additionally include event-year dummies to account for unobserved year-specific effects in the abnormal returns. We use robust, nonclustered (as we only have one observation per bank) standard errors. ε_{it} again represents the remaining error term.

The results of this analysis are shown in Table 2.4. Column 1 presents the first-stage output. Although this step is only used to calculate the respective inverse Mill's ratios (i.e., λ_i) for the second stage, it is interesting to observe that the peer effects significantly drive a bank's probability of announcing a digital platform strategy. Columns 2-5 display the results for the second stage of the Heckman correction. The insignificant coefficients for λ_i across all event windows suggest that our findings do not suffer from a potential self-selection bias.[4] Note that the Heckman correction assumes a bivariate normal distribution of the error terms in both stages; thus, we excluded eight outliers to comply with this assumption. Moreover, the Bloomberg database did not provide total assets for one bank in our sample, and we consequently had to exclude this bank. Therefore, the number of banks for this exercise decreases from 165 to 156.

[4] Note that we also estimated the second stage regression without the subsample dummy variables $Developed_i$ and $AIorientation_i$. In this alternative specification, coefficients for the inverse Mill's ratios remain insignificant across all event windows (not tabulated).

	First Stage	Second Stage			
**Event window**		**[0,2]**	**[0,3]**	**[-2,2]**	**[-3,3]**
λ	–	-0.821<br>(2.876)	-1.665<br>(2.972)	-0.299<br>(2.928)	-1.585<br>(2.427)
ln $(TotalAssets)$	0.025<br>(0.023)	–	–	–	–
$PeerEffect$	0.029**<br>(0.012)	–	–	–	–
$Developed$	–	-0.080<br>(0.201)	-0.078<br>(0.180)	-0.154<br>(0.188)	-0.103<br>(0.156)
$AIorientation$	–	0.164<br>(0.148)	0.043<br>(0.146)	0.092<br>0.141	-0.034<br>(0.130)
Time-fixed effects	Yes	Yes*	Yes*	Yes*	Yes*
N	815	156	156	156	156
R^2	0.330	0.056	0.059	0.079	0.119

Notes: As indicated in equation (9), ln $(TotalAssets)$ *is based on the year before the event. Robust standard errors are in parentheses. *** p < 0.01; ** p < 0.05; * p < 0.1.* Time-fixed effects in the second stage regressions represent event year dummies as indicated in equation (11). Pseudo-R^2 reported for the first stage regression.*

Table 2.4. Results of the Heckman Correction

Selection concerns in event studies have also been discussed in finance by Ahern [2]. The author shows that the statistical tests for event studies from a non-random sample can be biased. This bias occurs when firms in the sample are grouped by specific characteristics, for example, size, accounting ratios, or prior returns. Ahern [2] recommends using a so-called "characteristic-based benchmark model" instead of the classical market index to remedy this bias. The idea of this approach is that the underlying index resembles the firms analyzed in the event study. Since our sample might also represent a grouped sample as banks are similarly exposed to economic shocks (e.g., interest rates, macroeconomic news, etc.), we re-estimated the event study for the main effect hypothesized in Hypothesis 1 using an index that only comprises banks and related financial institutions (i.e., MSCI World Financials Index, Bloomberg ticker MXWO0FN Index). The results for this additional robustness test are shown in Table 2.5 and are qualitatively similar to the main analysis with the MSCI World Index, albeit less significant.

Event window	[0,1]	[0,2]	[0,3]	[-1,1]	[-2,2]	[-3,3]
CAAR (%)	0.12	0.38	0.45	0.12	0.55	1.01
p-value *t*-test	0.64	0.13	0.13	0.64	**0.09**	**0.01**
p-value Patell	0.78	0.38	0.31	0.78	0.30	**0.03**
p-value Patell (KP-adjusted)	0.81	0.44	0.38	0.81	0.36	**0.05**
p-value BMP	0.77	0.42	0.32	0.77	0.27	**0.01**
p-value BMP (KP-adjusted)	0.80	0.48	0.38	0.18	0.34	**0.03**
Share of positive CAR (%)	52.12	55.15	58.18	48.48	56.97	59.39

N = 165
CAAR = cumulative average abnormal returns
KP = Kolari and Pynnonen [21]
CAR = cumulative abnormal returns
BMP = Boehmer, Musumeci and Poulsen [4]

Table 2.5. Analysis of the Main Effect with the MSCI World Financials Index

Robustness Test – Cross-Sectional Regression on Abnormal Returns

In the main analysis, we assessed the moderating effects by comparing the cumulative abnormal average returns of the subsamples. To jointly examine the effects of the subsamples, we apply a cross-sectional regression analysis in which we regress the hypothesized moderating variables (*developed market* and *AI orientation*) as well as bank-specific control variables on cumulative standardized abnormal returns ($CSAR_i$). The estimation equation is specified as follows:

$$CSAR_i = \alpha + \beta_1 Developed_i + \beta_2 AIorientation_i + \beta_3\, ln(TotalAssets_i) + \beta_4 ln\,(1 + ROA_i) + \beta_5 ln\,(Leverage_i) + \beta_6 ln\,(Age_i) + \beta_7 EventYear + \varepsilon_i \quad (12)$$

where $CSAR_i$ is the cumulative standardized abnormal return for bank i during the event as calculated in Bose and Leung [6]. For this analysis, the variables of interest are $Developed_i$ and $AIorientation_i$: $Developed_i$ is a dummy variable being one if bank i is in the *developed market* subsample and zero otherwise. $AIorientation_i$ is a dummy being one if bank i is in the *AI orientation* subsample and zero otherwise. We refer to Table 2.6 for the variable description for the bank-specific control variables. We include event-year dummies to account for unobserved year-specific effects in the abnormal returns and use robust standard errors. ε_{it}

represents the remaining error term. We report the results for a quantile regression as applied in Bose and Leung [6] and for a standard OLS regression.

Variable	Description
$\ln\,(Age_i)$	Natural logarithm of age in years for bank i.
$\ln\,(TotalAssets_i)$	Natural logarithm of total assets (Bloomberg field BS_TOT_ASSET) for bank i in the event year.
$\ln(1 + ROA_i)$	Natural logarithm of return on assets (Bloomberg field RETURN_ON_ASSET) for bank i in the event year. Since few banks have a negative return on assets and the logarithm cannot be computed for negative values, we add a constant to all values in the sample [e.g., 34].
$\ln(Leverage_i)$	Natural logarithm of the ratio debt (Bloomberg field SHORT_AND_LONG_TERM_DEBT) to equity (Bloomberg field TOTAL_EQUITY) for bank i in the event year.

Table 2.6. Definition of Variables

The results of the cross-sectional regression analysis are shown in Panel A of Table 2.7 for the event windows [0,2] and [-3,3], respectively. While the moderating effect of developed markets is insignificant in both event windows, we observe a significant moderating effect for AI orientation in the event window [0,2] for one specification. Hence, there is some evidence that AI orientation has a significant moderating effect. Note that the Bloomberg database did not provide total assets for one bank in our sample (see also for the Heckman correction above) and, in addition, other financial control variables for one more bank in our sample. Consequently, we reduced the sample size from 165 to 163.[5]

Although the inclusion of bank-specific control variables in the regression analysis accounts for differences in bank characteristics, existing literature commonly applies propensity score matching to further reduce the risk of estimation biases that arise from such differences [e.g., 26, 33]. Consequently, we also applied a propensity score matching before the cross-sectional regression for additional robustness. We use kernel matching based on bank-specific variables [e.g., 18, 25] and apply the propensity score matching for both subsamples separately. After matching, the average reduction of standardized bias of covariates for developed market and AI

[5] When additionally applying the strict outlier exclusion as done for the Heckman correction above, the results remain qualitatively the same (not tabulated).

orientation are 50.3% and 71.7%, respectively. This reduction indicates that the matching procedures produced a better balance, yielding less bias in treatment effects estimation.

First, we created a matched sample for the development market subsample and estimated equation (12) above (Panel B of Table 2.7)[6]. The matching reduces our sample size to 141 (i.e., the excluded banks were too different to be matched with a counterpart). With the matched sample, a significant moderating effect of developed market emerges in the event window [-3,3] for one specification, and one more coefficient is at the brink of statistical significance (i.e., $p = 0.108$ for [0,2] in OLS). A nonparametric Wilcoxon rank-sum test further supports the effect for both event windows. The test shows a statistically significant difference (at the 10% level) between the standardized cumulative abnormal returns of the two matched groups of banks from developed vs. emerging markets.

Second, we created a matched sample for the AI orientation subsample and estimated equation (12) above (Panel C of Table 2.7). This matching reduces our sample size only slightly to 157 banks. This matching confirms the significance of the moderating effect of AI orientation for the event window [0,2]. Again, the effect is also identified as statistically significant at the 10% level with a Wilcoxon rank-sum test.

[6] For the regressions after the propensity score matching, we only include the dummy variable that has been used in the matching process to create to matched groups.

**Event window**	**[0,2]**		**[-3,3]**	
**Panel A**<br>N = 163 | no matching	OLS	Quantile	OLS	Quantile
$Developed$	-0.256<br>(0.252)	0.072<br>(0.182)	-0.213<br>(0.172)	-0.257<br>(0.262)
$AIorientation$	0.141<br>(0.204)	0.307**<br>(0.153)	-0.095<br>(0.220)	0.195<br>(0.147)
ln (Age)	0.076<br>(0.096)	0.110<br>(0.075)	0.030<br>(0.080)	-0.069<br>(0.070)
ln $(TotalAssets)$	-0.005<br>(0.035)	0.026<br>(0.030)	-0.007<br>(0.041)	-0.039<br>(0.031)
$\ln(1 + ROA)$	-0.049<br>(0.267)	0.098<br>(0.243)	-0.061<br>(0.216)	0.135<br>(0.203)
$\ln(Leverage)$	0.018<br>(0.062)	0.042<br>(0.047)	0.041<br>(0.056)	0.054<br>(0.042)
Event year dummies	Yes	Yes	Yes	Yes
R^2	0.025	0.064	0.016	0.082
**Panel B - Developed**<br>N = 141 | PSM	OLS	Quantile	OLS	Quantile
$Developed$	-0.446<br>(0.276)	-0.316<br>(0.295)	-0.319*<br>(0.185)	-0.317<br>(0.236)
ln (Age)	0.091<br>(0.106)	0.165<br>(0.096)	0.055<br>(0.084)	-0.048<br>(0.070)
ln $(TotalAssets)$	0.073<br>(0.044)	0.082<br>(0.035)	0.021<br>(0.037)	0.012<br>(0.031)
$\ln(1 + ROA)$	-0.082<br>(0.325)	-0.282<br>(0.256)	-0.225<br>(0.259)	-0.173<br>(0.208)
$\ln(Leverage)$	-0.044<br>(0.069)	-0.061<br>(0.065)	-0.003<br>(0.058)	0.004<br>(0.057)
Event year dummies	Yes	Yes	Yes	Yes
R^2	0.055	0.084	0.027	0.094
**Panel C - AI Orientation**<br>N = 157 | PSM	OLS	Quantile	OLS	Quantile
$AIorientation$	0.155<br>(0.199)	0.299*<br>(0.173)	-0.100<br>(0.215)	0.150<br>(0.166)
ln (Age)	0.079<br>(0.097)	0.107<br>(0.075)	0.024<br>(0.080)	-0.066<br>(0.070)
ln $(TotalAssets)$	0.033<br>(0.039)	0.052<br>(0.021)	-0.005<br>(0.043)	-0.010<br>(0.027)
$\ln(1 + ROA)$	0.051<br>(0.285)	0.092<br>(0.216)	-0.037<br>(0.215)	0.047<br>(0.195)
$\ln(Leverage)$	0.023<br>(0.069)	0.071<br>(0.051)	0.068<br>(0.065)	0.020<br>(0.051)
Event year dummies	Yes	Yes	Yes	Yes
R^2	0.022	0.075	0.011	0.079

Notes: As indicated in equation (12), financial accounting control variables are based on the year of the event. Robust standard errors are in parentheses. PSM = Propensity Score Matching. Adjusted R^2 reported for OLS, Pseudo-R^2 reported for Quantile regression. *** $p < 0.01$; ** $p < 0.05$; * $p < 0.1$.

Table 2.7. Results of the Cross-Sectional Regression with and without Propensity Score Matching

References (Appendix)

1. Agrawal, M.; R. Kishore, and H.R. Rao, Market reactions to E-business outsourcing announcements: An event study. *Information and Management*, 43, 7 (2006), 861-873.
2. Ahern, K.R., Sample selection and event study estimation. *Journal of Empirical Finance*, 16, 3 (2009), 466-482.
3. Bhagwat, Y.; N.L. Warren; J.T. Beck, and G.F. Watson, Corporate Sociopolitical Activism and Firm Value. *Journal of Marketing*, 84, 5 (2020), 1-21.
4. Boehmer, E.; J. Musumeci, and A. Poulsen, Event-study methodology under conditions of event-induced variance. *Journal of Financial Economics*, 30, 2 (1991), 253-272.
5. Bose, I. and A.C.M. Leung, Do phishing alerts impact global corporations? A firm value analysis. *Decision Support Systems*, 64 (2014), 67-78.
6. Bose, I. and A.C.M. Leung, Adoption of identity theft countermeasures and its short- and long-term impact on firm value. *MIS Quarterly*, 43, 1 (2019), 313-327.
7. Brown, S.J. and J.B. Warner, Using daily stock returns: The case of event studies. *Journal of Financial Economics*, 14, 1 (1985), 3-31.
8. Campbell, C.J.; A.R. Cowan, and V. Salotti, Multi-country event-study methods. *Journal of Banking & Finance*, 34, 12 (2010), 3078-3090.
9. Chai, S.; M. Kim, and H.R. Rao, Firms' information security investment decisions: Stock market evidence of investors' behavior. *Decision Support Systems*, 50, 4 (2011), 651-661.
10. Chatterjee, D.; C. Pacini, and V. Sambamurthy, The Shareholder-Wealth and Trading-Volume Effects of Information-Technology Infrastructure Investments. *Journal of Management Information Systems*, 19, 2 (2002), 7-42.
11. Chen, Y.; S. Ganesan, and Y. Liu, Does a firm's product-recall strategy affect its financial value? An examination of strategic alternatives during product-harm crises. *Journal of Marketing*, 73, 6 (2009), 214-226.
12. Dehning, B.; V.J. Richardson; A. Urbaczewski, and J.D. Wells, Reexamining the value relevance of e-commerce initiatives. *Journal of Management Information Systems*, 21, 1 (2004), 55-82.
13. Dehning, B.; V.J. Richardson, and R.W. Zmud, The Value Relevance of Announcements of Transformational Information Technology Investments. *MIS Quarterly*, 27, 4 (2003), 637-656.
14. Dos Santos, B.L.; K. Peffers, and D.C. Mauer, The Impact of Information Technology Investment Announcements on the Market Value of the Firm. *Information Systems Research*, 4, 1 (1993), 1-23.
15. Fama, E.F. and K.R. French, The Cross-Section of Expected Stock Returns. *The Journal of Finance*, 47, 2 (1992), 427-465.
16. Fama, E.F. and K.R. French. *Description of Fama/French 3 Factors for Developed Markets*. 2023; Accessed on February 15, 2023; Available from: http://mba.tuck.dartmouth.edu/pages/faculty/ken.french/Data_Library/f-f_3developed.html.
17. Fotheringham, D. and M.A. Wiles, The effect of implementing chatbot customer service on stock returns: an event study analysis. *Journal of the Academy of Marketing Science*, 51, 4 (2023), 802-822.
18. Ge, R.; J. Feng; B. Gu, and P. Zhang, Predicting and Deterring Default with Social Media Information in Peer-to-Peer Lending. *Journal of Management Information Systems*, 34, 2 (2017), 401-424.

19. Heckman, J.J., Sample selection bias as a specification error. *Econometrica: Journal of the Econometric Society*, 47, 1 (1979), 153-161.
20. Im, K.S.; K.E. Dow, and V. Grover, Research Report: A Reexamination of IT Investment and the Market Value of the Firm - An Event Study Methodology. *Information Systems Research*, 12, 1 (2001), 103-117.
21. Kolari, J.W. and S. Pynnonen, Nonparametric rank tests for event studies. *Journal of Empirical Finance*, 18, 5 (2011), 953-971.
22. MacKinlay, A.C., Event Studies in Economics and Finance. *Journal of Economic Literature*, 35, 1 (1997), 13-39.
23. Mani, D.; A. Barua, and A.B. Whinston, Outsourcing contracts and equity prices. *Information Systems Research*, 24, 4 (2013), 1028-1049.
24. Meng, Z. and S.Y.T. Lee, The value of IT to firms in a developing country in the catch-up process: An empirical comparison of China and the United States. *Decision Support Systems*, 43, 3 (2007), 737-745.
25. Mithas, S. and M.S. Krishnan, From association to causation via a potential outcomes approach. *Information Systems Research*, 20, 2 (2009), 295-313.
26. Oestreicher-Singer, G. and L. Zalmanson, Content or Community? A Digital Business Strategy for Content Providers in the Social Age. *MIS Quarterly*, 37, 2 (2013), 591-616.
27. Pacicco, F.; L. Vena, and A. Venegoni, From common to firm-specific event dates: A new version of the estudy command. *Stata Journal*, 21, 1 (2021), 141-151.
28. Patell, J.M., Corporate forecasts of earnings per share and stock price behavior: Empirical test. *Journal of Accounting Research*, 14, 2 (1976), 246-276.
29. Patell, J.M. and M.A. Wolfson, Anticipated information releases reflected in call option prices. *Journal of Accounting and Economics*, 1, 2 (1979), 117-140.
30. Ranganathan, C. and C.V. Brown, ERP investments and the market value of firms: Toward an understanding of influential ERP project variables. *Information Systems Research*, 17, 2 (2006), 145-161.
31. Subramani, M. and E. Walden, The Impact of E-Commerce Announcements on the Market Value of Firms. *Information Systems Research*, 12, 2 (2001), 135-154.
32. The World Bank. *World Bank Country and Lending Groups*. 2023; Accessed on Mai 16, 2023; Available from: https://datahelpdesk.worldbank.org/knowledgebase/articles/906519-world-bank-country-and-lending-groups.
33. Wang, S.A.; M.-S. Pang, and P.A. Pavlou, Cure or Poison? Identity Verification and the Posting of Fake News on Social Media. *Journal of Management Information Systems*, 38, 4 (2022), 1011-1038.
34. Wicklin, R., *Log transformations: How to handle negative data values?*, in *SAS Blogs*. 2011.
35. Wolfolds, S.E. and J. Siegel, Misaccounting for endogeneity: The peril of relying on the Heckman two-step method without a valid instrument. *Strategic Management Journal*, 40, 3 (2019), 432-462.
